# Role of a genetic variation in the microRNA-4421 binding site of *ERP29* regarding risk of oropharynx cancer and prognosis

**DOI:** 10.1038/s41598-020-73675-z

**Published:** 2020-10-12

**Authors:** Juliana Carron, Ana Paula Dalla Costa, José Augusto Rinck-Junior, Fernanda Viviane Mariano, Benilton de Sá Carvalho, Carmen Silvia Passos Lima, Gustavo Jacob Lourenço

**Affiliations:** 1 Laboratory of Cancer Genetics, School of Medical Sciences, University of Campinas, Rua Vital Brasil, 50, Distrito de Barão Geraldo, Campinas, São Paulo CEP: 13083-888 Brazil; 2 Department of Internal Medicine, School of Medical Sciences, University of Campinas, Campinas, São Paulo Brazil; 3 Department of Pathology, School of Medical Sciences, University of Campinas, Campinas, São Paulo Brazil; 4 Department of Statistics, Institute of Mathematics, Statistics and Scientific Computing, University of Campinas, Campinas, São Paulo Brazil

**Keywords:** Cancer genetics, Head and neck cancer

## Abstract

We conducted a two-stage association study on patients with oropharynx (OP) squamous cell carcinoma (SCC) and healthy controls to identify single nucleotide variants (SNVs) located at the microRNA (miR)-binding sites of carcinogenesis genes associated with risk and prognosis of the disease. In stage 1, 49 patients and 49 controls were analyzed using Genome-Wide Human SNV Arrays to identify variants in the 3′-untranslated region (3′-UTR) of carcinogenesis-related genes, and one SNV was selected for data validation in stage 2 by TaqMan assays in 250 OPSCC patients and 250 controls. The *ERP29* c.*293A > G (rs7114) SNV located at miR-4421 binding site was selected for data validation among 46 SNVs. The ERp29 and miR-4421 levels were evaluated by quantitative-PCR and Western blotting. Interaction between miR-4421 with 3′-UTR of *ERP29* was evaluated by luciferase reporter assay. Event-free survival (EFS) was calculated by Kaplan–Meier and Cox methods. *ERP29* GG variant genotype was more common in OPSCC patients than in controls (6.4% *vs* 3.6%, *p* = 0.02; odds ratio: 5.67; 95% confidence interval (CI) 1.27–25.26). Shorter EFS were seen in the base of tongue (BT) SCC patients with GG genotype (0.0% *vs* 36.2%, *p* = 0.01; hazard ratio: 2.31; 95% CI: 1.03–5.15). Individuals with *ERP29* AG or GG genotypes featured lower levels of *ERP29* mRNA (*p* = 0.005), ERp29 protein (*p* < 0.001) and higher levels of miR-4421 (*p* = 0.02). The miR-4421 showed more efficient binding with 3′-UTR of the variant G allele when compared with wild-type allele A (*p* = 0.001). Our data suggest that *ERP29* rs7114 SNV may alter the risk and prognosis of OPSCC due to variation in the ERp29 production possibly modulated by miR-4421.

## Introduction

Smoking habit and alcohol consumption consist in the classical risk factors for developing oropharynx (OP) squamous cell carcinoma (SCC)^1^. Sexual behavior is also established as a risk factor for human papillomavirus (HPV)-related OPSCC^2^. Most of OPSCC patients are diagnosed with measurable locally advanced disease^3^, and only about half of these patients achieve complete or partial responses after 5-years survival^4^.

Endoplasmic reticulum (ER) protein 29 (p29) is a chaperone protein that functions in unfolding and facilitating transport of synthesized secretory proteins from the ER to Golgi^5^. Its function in cancer has been actively addressed, but its role in cancer development and progression is still unclear^6^. The ERp29 expression was found to be inversely associated with tumor development in the lung^7^, breast^8^, and gallbladder^9^; moreover, it inhibited breast tumor formation in mice^10^. In contrast, ERp29 overexpression was observed in breast, melanoma, lung, cervical^11^, liver^12^, and metastatic colorectal^13^ cancer cell line.

Additionally, ERp29 was found to regulate breast cancer cell growth arrest through p38 activation and upregulation of the ER stress protein p58^IPK^^14^, and cancer cell survival against genotoxic stress induced by doxorubicin^15,16^, cisplatin (CDDP)^17^, gemcitabine^18^, and radiation^19,20^. ERp29 overexpression was associated with unfavorable prognosis of colorectal cancer through activation of chloride intracellular channel 4 and second mitochondria-derived activator of caspases proteins^13^. On the other hand, better prognosis was observed in pancreatic ductal adenocarcinoma^21^ and gastric cancer^22^ with ERp29 overexpression.

Noteworthily, ERp29 overexpression was associated with mesenchymal-epithelial transition (MET) upregulation and epithelial morphogenesis^8–10^ as well as with epithelial-mesenchymal transition downregulation in cancer cells^23^. In fact, ERp29 overexpression was showed to be associated with metastasis promotion in breast cancer^24^, uveal melanoma^25^, and colorectal cancer^26^. However, there are no studies focusing on the role of ERp29 in OPSCC risk or prognosis.

In addition, genetic association studies have identified single nucleotide variants (SNVs) related to OPSCC risk and prognosis^27^. Several SNVs previously identified are located in non-coding regions of the genome, including the 3′-untranslated region (3′-UTR)^27^. SNVs located in the 3′-UTR of genes could influence microRNAs (miR) binding and direct posttranscriptional repression of genes involved in carcinogenesis, including the OPSCC^28^. Besides, SNVs can affect the ability of a protein to fold and remain stable inside cells, often leading to diseases^29^.

The population of Brazil is highly heterogeneous and admixed, as a result of cross-breeding among native Amerindians, Europeans settlers and immigrants, and sub-Saharan Africans^30^. Since SNVs in genes with importance in OPSCC risk may not have been selected in previous studies, we conducted a two-stage association study on patients with OPSCC and healthy controls from the Southeast region of Brazil identifying a SNV in the *ERP29* (c.*293A > G, rs7114) associated with risk of tumor and prognosis. Moreover, we found that it is a functional SNV that alters the binding of miR-4421 and ERp29 levels.

## Material and methods

### Study population

This study was conducted in two stages. In stage 1, 49 patients and 49 controls were analyzed with the purpose of identifying SNVs on miR-binding sites of carcinogenesis genes with importance in OPSCC risk, and in stage 2, one SNV was selected for data validation in 250 OPSCC patients and 250 controls.

All OPSCC patients were diagnosed at the Clinical Oncology Service of the University of Campinas Teaching Hospital between June 2000 and April 2016. The control group comprised 250 blood donors of the same sex and ethnicity from the same Teaching Hospital. All subjects were classified as either smokers and non-smokers, and drinkers or abstemious, as previously reported^31^. The Institutional Research Committee of University of Campinas approved the study (numbers: 424/2016 and 1.438.601). All procedures were carried out according to the Helsinki Declaration, and appropriate informed consent form was obtained.

OPSCC was diagnosed according to World Health Organization criteria^32^. Histologically, the OPSCC was classified as well, moderately, poorly differentiated, or undifferentiated^33^. In addition, the OPSCC was staged according to the TNM system of the 7th American Joint Committee of Cancer Staging^34^.

HPV status was performed in available tumor fragments embedded in paraffin of 98 OPSCC patients. We could not obtain the HPV status from 152 patients due to unavailable tumor fragments. P16 immunohistochemistry and in situ hybridization were performed in tumor fragments, aiming to test the presence of human papillomavirus type 16 (HPV 16)^35^.

For survival analysis, we selected 226 patients; 24 out of 250 patients were sent to other services for treatment and follow-up, and no consistent clinical information could be obtained. Patients were treated according to the institutional protocol, based on conventional procedures^31^. Patients with locoregional advanced resectable tumors received neoadjuvant treatment (n = 19) or adjuvant treatment (n = 36) with 35 sessions of radiation, 2 Gy per session, with concurrent intravenous CDDP at a dose of 80–100 mg/m^2^ or carboplatin area under the curve of 5 on days 1, 22, and 43, before or after surgery, respectively. For 168 patients, definite treatment with 35 sessions of radiation, 2 Gy per session, and concurrent intravenous CDDP or carboplatin at aforementioned doses, was administered, to whom surgical treatment was not performed because of locoregional unresectable tumors, low Karnofsky performance scale score, refusal of surgery due to expected functional or anatomic sequels, or an organ preservation protocol. We could not obtain treatment information from 3 patients enrolled in survival analysis due to lack of consistent clinical information. Patients’ follow-up was performed at 3-month intervals. The end of the follow-up period was January 2019.

### Stage 1: screening of SNVs, candidate gene choice and SNV selection

Ninety-eight individuals were analyzed in the first stage. Genomic DNA from peripheral blood of 49 base of tongue (BT) SCC patients and 49 controls was genotyped for a total of 500,568 SNVs using the Affymetrix Genome-Wide Human SNV Array 5.0 (AFFYMETRIX, USA) according to the manufacturer’s recommended protocols. The intensities resulting from the arrays scanning process were made available via CEL files, one per DNA sample with total quality control higher than 90% (AFFYMETRIX, USA). Tools from the Bioconductor (www.bioconductor.org) were used to process the CEL files. The genotyping was performed applying the corrected robust linear mixture model (crlmm) algorithm^36^.

After association analysis (patients *vs* controls), SNVs located in the 3′-UTR of genes previously reported to be associated with, or known to be involved in carcinogenesis pathways, were selected. The analysis of carcinogenesis pathways was performed using The Database for Annotation, Visualization and Integrated Discovery^37^ and Kyoto Encyclopedia of Genes and Genomes pathway maps^38^.

SNVs showing significant deviation from Hardy–Weinberg (HW) equilibrium in controls, and those with minor allele frequency less than 10%, were excluded from the selection. SNV selection across each of carcinogenesis-related genes was carried out calculating their sample size based on the genotypic frequencies observed in healthy individuals from different ethnic populations^39^ and also using MicroSNiPer^40^ and MirSNPscore^41^ algorithms to select variations in miRNA binding sites sequences of 3′-UTR. We select the SNVs that presented a sample size less or equal than 250 individuals, which is the total patients’ sample available in our biorepository. We selected the miRNAs that match six, seven, or eight nucleotides in seed region (position 2–8 of miRNA). The RNAhybrid software^42^ was used for finding the energetically most favorable hybridization sites using the minimum free energy (MFE) of hybridization of − 20 kcal/mol or less. As a result, in this study we selected the *ERP29* rs7114 for further validation.

### Stage 2: validation of SNV *ERP29* rs7114 in OPSCC risk

Five hundred individuals were analyzed in the second stage, including the 98 individuals analyzed preliminary in stage 1. Genomic DNA from leukocytes of peripheral blood of 250 OPSCC patients and 250 controls was analyzed by real-time polymerase chain reaction (PCR), using TaqMan SNV genotyping assay (assay reference: C_7521976_10, APPLIED BIOSYSTEMS, USA) for *ERP29* rs7114 genotyping, according to the manufacturer’s instructions. The amount of 20% of genotype determination was carried out twice in independent experiments with 100% agreement.

### *ERP29* expression by quantitative PCR

Total RNA from leukocytes of peripheral blood of 55 healthy individuals with distinct genotypes of *ERP29* (22 individuals with AA, 23 with AG and 10 with GG genotypes) was extracted with TRIzol reagent (LIFE TECHNOLOGIES, USA), according to the manufacturer’s instructions. cDNA was generated using SuperScript III reagents (LIFE TECHNOLOGIES, USA). Experiments were performed using SYBR Green PCR Master Mix reagents (APPLIED BIOSYSTEMS, USA) and specific primers for the *ERP29* gene (forward: 5′-CAGAGGTGGGGATCTCAGATTAT-3′, and reverse: 5′-GAAGACTGGGTAGCTCTCTTTGTC-3′), in triplicate per sample, and a negative control without template was included in each plate. The relative expression level of *ERP29* was normalized to the reference housekeeping gene actin beta level (forward: 5′-AGGCCAACCGCGAGAAG-3′, and reverse: 5′-ACAGCCTGGATAGCAACGTACA-3′) using the 2^−ΔΔCt^ cycle threshold method. Values of 20% of the samples were repeated in separate experiments with 100% agreement. Results were expressed in arbitrary units (AUs).

### miR-4421 expression

Specific cDNA for miR-4421 and RNU24 (endogenous control) were generated using TaqMan MicroRNA Reverse Transcription kit (LIFE TECHNOLOGIES, USA), according to the manufacturer’s instructions. The cDNA (n = 44) was obtained from previously extracted total RNA (14 individuals with AA genotype of *ERP29*, 18 individuals with AG and 12 individuals with GG). Reactions of qPCR were performed using TaqMan Universal PCR Master Mix II, no UNG (LIFE TECHNOLOGIES, USA) and TaqMan MicroRNA Assay RT-PCR (miR-4421: assay ID 464860_mat and RNU24: assay ID: 001001; LIFE TECHNOLOGIES, USA), in triplicate per sample, and a negative control without cDNA was included in all plate reactions. The relative expression level of miR-4421 was normalized to miRNA RNU24 using the 2^−ΔΔCt^ cycle threshold method. Results were expressed in AUs.

### Western blotting

Leukocytes of peripheral blood of 28 healthy individuals with distinct *ERP29* genotypes (15 individuals with AA genotype, 10 individuals with AG and 3 with GG) were used for extraction of total proteins. Briefly, cells were lysed with RIPA buffer containing protease inhibitors. Total protein concentrations were measured by Lowry protein assay. The cell lysates (40 µg) were subjected to 12% SDS-PAGE, and proteins were transferred to nitrocellulose membranes. The proteins reacted with rabbit anti-ERp29 monoclonal antibody (1:2000, ab176573; ABCAM, GBR) and rabbit anti-glyceraldehyde-3-phosphate dehydrogenase (GAPDH) (1:1000, sc-47724; SANTA CRUZ, GBR) overnight at 4 °C. A horseradish peroxidase-conjugated goat anti-rabbit IgG antibody was used as the secondary antibody (1:10,000, ab97051; ABCAM, GBR). Chemiluminescent signals were visualized in the capture imaging system ImageQuant 350 (GE HEALTHCARE, SWE) using SuperSignal West Pico PlusChemiluminescent Substrate (THERMO SCIENTIFIC, USA), and signal intensity was analyzed by the ImageJ software (NATIONAL INSTITUTES OF HEALTH, USA). The level of GAPDH was used as loading control.

### Human pharynx SCC cell line culture

The human pharynx SCC cell line (FaDu) (ATCC HTB-43) was cultured in Dulbecco’s modified Eagle’s (DMEM) medium (GIBCO, USA) supplemented by 10% fetal bovine serum (FBS) and 100 μg/ml penicillin–streptomycin (SIGMA-ALDRICH, DEU) in an incubator at 37 °C with humidified atmosphere of 5% CO_2_. FaDu cell line was authenticated using short tandem repeat analysis^43^. All experiments were performed with mycoplasma-free cells.

### Construction of plasmids

The 3′-UTR of *ERP29* rs7114_A (wild-type allele) and rs7114_G (variant allele) mRNA (266 bps) of individuals with known *ERP29* AA and GG genotypes, respectively, were amplified by PCR using 2U of Platinum Taq DNA Polymerase High Fidelity (THERMO SCIENTIFIC, USA) and specific primers with restriction site for *Spe*I (forward: 5′-GCACTAGTCTTGGGATGTCTCTAGCTGG-3′; where the *Spe*I site is underlined) and *Mlu*I (reverse: 5′-ATACGCGTATACCAGCTTAGATTCAAAG-3′, where the *Mlu*I site is underlined). Fragments were cloned into the pMIR-REPORT miRNA Expression Reporter Vector (AMBION, USA) immediately downstream of the firefly luciferase gene driven by the CMV promoter, using standard protocols. After procedures, the plasmids pMIR_rs7114_A and pMIR_rs7114_G were obtained.

### Dual luciferase reporter assay

FaDu cells were transiently transfected with the plasmids pMIR_rs7114_A, pMIR_rs7114_G, *Renilla* luciferase control reporter (pRL) (normalizing control) (PROMEGA, USA); and synthetic sequences of miR-4421 mimics and inhibitor mimics (AMBION, USA), using Lipofectamine 2000 (INVITROGEN, USA), according to the manufacturer’s instructions. FaDu cells were seeded in DMEM medium 48 h prior to transfection. In summary, 2 × 10^5^ cells were transfected in four different groups: (1) 10 ng of pMIR_rs7114_A co-transfected with 5 ng of pRL and 50 nM of miR-4421; (2) 10 ng of pMIR_rs7114_A co-transfected with 5 ng of pRL and 50 nM of miR-4421 inhibitor; (3) pMIR_rs7114_G co-transfected with 5 ng of pRL and 50 nM of miR-4421 and; (4) pMIR_rs7114_G co-transfected with 5 ng of pRL and 50 nM of miR-4421 inhibitor. Cells of each group were plated in reduced serum medium Opti-MEM (GIBCO, USA) for 6 h; then, DMEM medium containing 2% FBS and 100 μg/ml penicillin–streptomycin (SIGMA-ALDRICH, DEU) was added. Cells were harvested at 48 h after transfection and luciferase activity was measured using the Dual-Luciferase Reporter Assay System kit (PROMEGA, USA), according to the manufacturer’s instructions. Relative firefly luciferase activity was normalized to the pRL vector activity. Assays were performed in triplicate, repeated, and included a negative control in each reaction.

### Statistical analysis

Association between disease statuses, BTSCC patients *vs* controls, and genotypes for stage 1 was performed using the logistic regression model. These analyses were adjusted by age at diagnosis, sex and skin color. SNVs that presented raw *p*-values below the 0.01 thresholds were selected for further inspection. These analyses were implemented in R software (www.r-project.org).

The HW equilibrium was tested using chi-square (χ^2^) statistics for the goodness-of-fit test. Differences between groups were analyzed by χ^2^ or Fisher’s exact test. Multivariate analysis using logistic regression model served to obtain age- and tobacco status-adjusted crude odds ratios (ORs) with 95% confidence intervals (CI), and to assess associations between genotypes and OPSCC in stage 2. Power of analysis (PA) was used to calculate the minimum effect size that is likely to be detected in a study using a given sample size (DSS Research: https://bit.ly/2Fe79sl). χ^2^ and Fisher’s exact tests were used to evaluate possible associations between clinical characteristics, tumor aspects, and the genotypes of the selected SNV. Considering continuous variables, data sets were probed for normality using Shapiro–Wilk’s test. For the *ERP29* gene expression, the data set assumed normal distribution and *t* test was used for analysis. For ERp29 protein content, miR-4421 expression and luciferase assay, data sets did not assume normal distribution, thus, we used the Mann–Whitney test to compare the groups.

For survival analysis, the event-free survival (EFS) was calculated from the date of diagnosis until the date of progression of disease, the first relapse, death by disease, or the last follow-up. Overall survival (OS) was calculated from the date of diagnosis until the date of death, resulting from any cause, or the date of last follow-up. EFS and OS times were calculated using Kaplan–Meier (K–M) estimate probabilities, and differences between survival curves were analyzed by the log-rank test^44^. The prognostic impact of age at diagnosis, sex, histological grade, TNM stage and *ERP29* genotypes in survival of OPSCC patients was examined using Cox proportional hazard ratio (HR) regression. In a second step, all variables with *p* < 0.15 were included in a multivariate Cox regression (backward conditional step wise selection)^44^. For all statistical tests, significance is two-sided and achieved when *p-*values were ≤ 0.05. Tests were done using the SPSS 21.0 software (SPSS INCORPORATION, USA).

## Results

### Study population

We present demographic data and smoking and alcohol habits of 250 OPSCC patients and 250 controls in Table S1 Supplement. Control individuals were younger than OPSCC patients (median age: 44 *vs* 56 years, *p* < 0.001), and the number of tobacco and alcohol users was higher among patients than in controls (89.6% *vs* 13.2%, *p* < 0.001; 78.8% *vs* 49.2%, *p* < 0.001; respectively). Differences in age and pattern of tobacco and alcohol habits of individuals of each group were corrected in all comparisons of genotype frequencies by pertinent statistical analyses.

We show frequencies of tumor characteristics of OPSCC patients in Table S2 Supplement. Most patients presented tumors located in the BT (46.4%), moderately differentiated tumors (64.8%), and advanced tumor stage (IV) (72.8%). HPV type 16 was positive in only 8 out of 98 OPSCC patients analyzed in the study.

Patients were treated with CDDP, radiotherapy (RT), and surgery. One hundred and sixty eight patients (68.6%) were submitted to chemotherapy (CT) and RT combined treatment; 47 patients (19.2%) received CT, RT, and were submitted to surgery; 12 patients (4.9%) received RT and surgery; 7 patients (2.9%) only received CT; 6 patients (2.4%), only RT; and 5 patients (2.0%) were only submitted to surgery. We could not obtain consistent information about the therapeutics of 5 patients.

### Stage 1: analysis of screening of SNVs

We present clinical and pathological characteristics of the 98 participants included in stage 1 in Table S3 Supplement. After screening of SNVs (49 BTSCC patients *vs* 49 controls), we observed 6,609 SNVs associated with risk of BTSCC; 3461 (52.4%) of them were located in regulatory regions; 3,045 (46.0%), in introns; 52 (0.8%), in coding regions; 46 (0.7%), in 3′-UTR; and 5 (0.1%) SNVs, in 5′-UTR. Data on the genome association were deposited at the Gene Expression Omnibus database (www.ncbi.nlm.nih.gov/geo/) with accession number GSE46812.

### Stage 1: SNV selection

**Figure 1:**
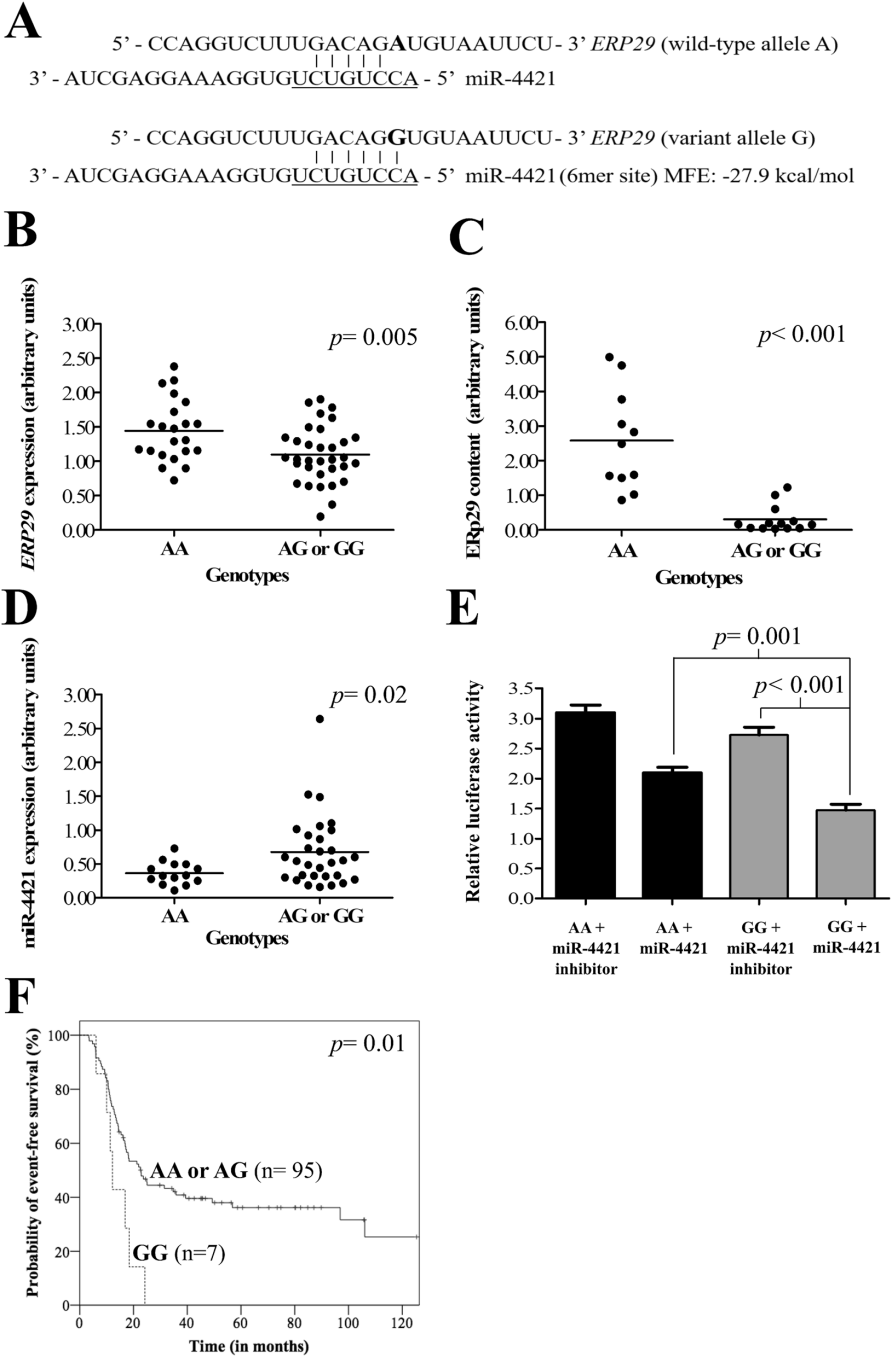
*ERP29* rs7114 single nucleotide variation (SNV) modulated gene expression and protein content, possible due to miR-4421 affinity. (**A**) Predicted microRNA (miRNA) miR-4421 binding site in *ERP29* 3′-unstranslated region (3′-UTR) related to rs7114 SNV. The miRNA “seed” region is presented in underline font. The rs7114 SNV of *ERP29* is represented in bold letter. The variant allele G creates a binding site of six nucleotides (6mer site) to miR-4421. The wild-type allele A disrupts the binding site. (**B**) *ERP29* rs7114 genotypes and gene expression. The mean mRNA expression level was lower in individuals with *ERP29* AG or GG (*p* = 0.005) when compared with the AA genotype. (**C**) *ERP29* rs7114 genotypes and protein level by Western blotting. The ERp29 protein content was lower in individuals with *ERP29* AG or GG genotypes (*p* < 0.001) when compared with the AA genotype. (**D**) *ERP29* rs7114 genotypes and miRNA miR-4421 expression. The mean miR-4421 expression level was higher in individuals with *ERP29* AG or GG (*p* = 0.02) when compared with the AA genotype. (**E**) Luciferase activity in different groups: (1) pMIR-*ERP29*_AA (*ERP29* rs7114 AA genotype) co-transfected with miR-4421 inhibitor; (2) pMIR-*ERP29*_AA co-transfected with miR-4421 mimics; (3) pMIR-*ERP29*_GG (*ERP29* rs7114 GG genotype) co-transfected with miR-4421 inhibitor; and (4) pMIR-*ERP29*_GG co-transfected with miR-4421 mimics, in pharynx squamous cell carcinoma cell line, FaDu (ATCC). (*) FaDu cells co-transfected with pMIR-*ERP29*_GG and miR-4421 mimics presented lower luciferase activity when compared with those co-transfected with pMIR-*ERP29*_AA and miR-4421 mimics (*p* = 0.001). (**) FaDu cells co-transfected with pMIR-*ERP29*_GG and miR-4421 inhibitor featured an increase in luciferase activity when compared with those co-transfected with pMIR-*ERP29*_GG and miR-4421 mimics (*p* < 0.001). (**F**) Probability of event-free survival (EFS) of 102 base of tongue squamous cell carcinoma patients stratified by *ERP29* rs7114 SNV genotypes. The Kaplan–Meier curve indicates lower EFS in patients with the GG variant genotype (0.0% *vs* 36.2%, *p* = 0.01) when compared with patients with AA or AG genotypes. All statistical data analyses were performed using SPSS version 21.0 (www.ibm.com/analytics/spss-statistics-software).

After the SNVs screening, 16 of 46 SNVs located in the 3′-UTR were related to genes of carcinogenesis pathways (Table S4 Supplement). Only six of the 16 SNVs presented the appropriate sample size (n = 250): *JMJD6* rs2240774, *SLCO2A1* rs2370512, *SLC7A11* rs7674870, *MYO6* rs6914716, *TUSC1* rs1128957 and *ERP29* rs7114 (Table S4 Supplement). Among them, we select the SNV *ERP29* rs7114 for further validation in stage 2. We found that miR-4421 (MFE: − 27.9 kcal/mol) (Fig. 1A) matched 6mer site complementary in seed sequence of 3′-UTR of variant allele G of *ERP29* (rs7114), while the wild-type allele A disrupted these target sites.

### Stage 2: analysis of validation of *ERP29* rs7114

Samples of 250 OPSCC patients (χ^2^ = 0.82, *p* = 0.36) and 250 controls (χ^2^ = 1.10, *p* = 0.29) were in HW equilibrium at the *ERP29* rs7114 locus. *ERP29* GG variant genotype (6.4% *vs* 3.6%, *p* = 0.02) and G allele (23.2% *vs* 16.4%, *p* = 0.02) were more common in OPSCC patients than in controls. Individuals with GG genotype were under 5.67-fold increased risk of OPSCC than those with the remaining genotypes (Table 1). Moreover, individuals carrying at least one variant allele G were under 2.13-fold increased risk of OPSCC than carriers of wild-type A allele (Table 1).

**Table 1:** *ERP29* rs7114 genotypes in 250 oropharynx squamous cell carcinoma and 250 controls.

Genotypes	OPSCC n (%)	Controls n (%)	OR (95% CI)	*p*-value
***ERP29 rs7114***				
AA	150 (60.0)	177 (70.8)	Reference	
AG	84 (33.6)	64 (25.6)	1.69 (0.83–3.43)	0.14
GG	16 (6.4)	9 (3.6)	5.67 (1.27–25.26)*	**0.02**
AA or AG	234 (93.6)	241 (96.4)	Reference	
GG	16 (6.4)	9 (3.6)	5.50 (1.19–25.38)**	**0.02**
AA	150 (60.0)	177 (70.8)	Reference	
AG or GG	100 (40.0)	73 (29.2)	2.05 (1.04–4.05)***	**0.03**
A allele	384 (76.8)	418 (83.6)	Reference	
G allele	116 (23.2)	82 (16.4)	2.13 (1.20–3.78)****	**0.02**

*OPSCC* oropharynx squamous cell carcinoma, *n* number of patients or controls, *OR* odds ratio adjusted by age, smoking and drinking status, *CI* confidence interval.

The significant values are indicated by bold letters.

*Power of analysis (PA): 30.0%; **PA: 30.0%; ***PA: 72.0%; ****PA: 77.1%.

### Association between clinical and tumorous aspects with *ERP29* genotypes

No associations between *ERP29* rs7114 genotypes were seen in OPSCC patients stratified by age, sex, tobacco and alcohol consumption, histological grade, tumor stage, and tumor localization (Table 2).

**Table 2:** Association of *ERP29* rs7114 genotypes, clinical and tumor characteristics of 250 oropharynx squamous cell carcinoma patients.

Characteristics	n	*ERP29* rs7114							
		AA	AG	GG	AA or AG		GG	AA	AG or GG
**Sex**	250								
Male	228	135 (59.2%)	79 (34.6%)	14 (6.2%)	214 (93.8%)		14 (6.2%)	135 (59.2%)	93 (40.8%)
Female	22	15 (68.2%)	5 (22.7%)	2 (9.1%)	20 (90.9%)		2 (9.1%)	15 (68.2%)	7 (31.8%)
* p-*value		Reference	0.33	0.67	Reference		0.63	Reference	0.49
**Tobacco consumption**	250								
Smokers	224	135 (60.3%)	76 (33.9%)	13 (5.8%)	211 (94.2%)		13 (5.8%)	135 (60.3%)	89 (39.7%)
Non-smokers	26	15 (57.7%)	8 (30.7%)	3 (11.6%)	23 (88.5%)		3 (11.5%)	15 (57.7%)	11 (42.3%)
*p-*value		Reference	1.00	0.38	Reference		0.22	Reference	0.83
**Alcohol consumption**	250								
Drinkers	197	118 (59.9%)	69 (35.0%)	10 (5.1%)	187 (94.9%)		10 (5.1%)	118 (59.9%)	79 (40.1%)
Abstainers	53	32 (60.4%)	15 (28.3%)	6 (11.3%)	47 (88.7%)		6 (11.3%)	32 (60.4%)	21 (39.6%)
*p-*value		Reference	0.61	0.20	Reference		0.11	Reference	1.00
**Histological grade**	211*								
Well or moderately	176	104 (59.1%)	59 (33.5%)	13 (7.4%)	163 (92.6%)		13 (7.4%)	104 (59.1%)	72 (40.9%)
Poorly or undifferentiated	35	19 (54.3%)	14 (40.0%)	2 (5.7%)	33 (94.3%)		2 (5.7%)	19 (54.3%)	16 (45.7%)
*p-*value		Reference	0.55	1.00	Reference		1.00	Reference	0.70
**Tumor stage**	245*								
I or II	21	10 (47.6%)	11 (52.4%)	0 (0.0%)	21 (100.0%)		0 (0.0%)	10 (47.6%)	11 (52.4%)
III or IV	224	137 (61.5%)	71 (31.4%)	16 (7.1%)	208 (92.9%)		16 (7.1%)	137 (61.5%)	87 (38.5%)
*p-*value		Reference	0.15	0.60	Reference		0.37	Reference	0.25
**Tumor localization**	245**								
Base of tongue	116	67 (57.8%)	41 (35.3%)	8 (6.9%)	108 (93.1%)		8 (6.9%)	67 (57.7%)	49 (42.3%)
Tonsillar complex	84	52 (61.9%)	26 (30.9%)	6 (7.2%)	78 (92.9%)		6 (7.1%)	52 (61.9%)	32 (38.1%)
Soft palate	45	27 (60.0%)	16 (35.5%)	2 (4.5%)	43 (95.5%)		2 (4.5%)	27 (60.0%)	18 (40.0%)
*p-*value		Reference	0.80	0.83	Reference		0.82	Reference	0.83

*n* number of patients.

*The number of patients differed from the total quoted in the study (n = 250), because it was not possible to obtain consistent information about histological grade and tumor stage in some cases.

**The number of patients differed from the total quoted in the study (n = 250), because it was considered for analysis only the most frequent tumor localization.

### *ERP29* expression

Lower mRNA expression levels were seen in individuals with *ERP29* AG (n = 23) (1.17 AUs ± standard deviation (SD): 0.45, *p* = 0.04) and GG (n = 10) (0.94 AUs ± SD: 0.25, *p* = 0.002) than in those with the AA genotype (n = 22) (1.44 AUs ± SD: 0.45). In addition, individuals with *ERP29* AG or GG genotypes (n = 33) presented lower mRNA levels than those with the AA genotype (n = 22) (1.10 AUs ± 0.41 SD *vs* 1.44 AUs ± 0.45 SD, *p* = 0.005) (Fig. 1B).

### ERp29 protein content

Lower ERp29 protein content was observed in individuals with *ERP29* AG (n = 16) (0.79 AUs ± SD: 0.48, *p* < 0.001) and GG (n = 4) (0.98 AUs ± SD: 0.58, *p* = 0.03) than in those with the AA genotype (n = 11) (2.58 AUs ± SD: 1.44). In addition, individuals with *ERP29* AG or GG genotypes (n = 20) presented lower ERp29 protein content than those with the AA genotype (n = 11) (0.83 AUs ± 0.48 SD *vs* 2.58 AUs ± 1.44 SD, *p* < 0.001) (Fig. 1C).

### miR-4421 expression

Higher miR-4421 expression levels were observed in individuals with *ERP29* AG (n = 18) (0.60 AUs ± SD: 0.28, *p* = 0.01) than in those with the AA genotype (n = 14) (0.36 AUs ± SD: 0.16). Similar miR-4421 expression levels were perceived in individuals with *ERP29* GG (n = 12) (0.79 AUs ± SD: 0.76, *p* = 0.21) than in those with the AA genotype (n = 14) (0.36 AUs ± SD: 0.16). However, individuals with *ERP29* AG or GG genotypes (n = 30) presented higher miR-4421 expression than those with the AA genotype (n = 14) (0.67 AUs ± 0.52 SD *vs* 0.36 AUs ± 0.16 SD, *p* = 0.02) (Fig. 1D).

### Luciferase reporter assay

FaDu cells co-transfected with pMIR_rs7114_G (containing variant homozygous GG genotype) and miR-4421 mimics featured lower luciferase activity when compared with those co-transfected with pMIR_rs7114_A and miR-4421 mimics (*p* = 0.001) (Fig. 1E). Besides, FaDu cells co-transfected with pMIR_rs7114_G and miR-4421 inhibitor featured an increase in luciferase activity when compared with those co-transfected with pMIR_rs7114_A and miR-4421 mimics (*p* < 0.001) (Fig. 1E).

### Survival analysis of OPSCC patients

We obtained consistent survival data from 226 OPSCC patients. The median follow-up of patients enrolled in survival analysis was 33.3 months (range 1.5–162.2 months). The final status of patients was established on January 2019. At this time, 60 patients were alive without disease, 6 patients were alive with disease, 123 patients died due to disease, and 37 patients died due to unrelated causes. The 5-year EFS and OS rates were 42.8% and 38.7%, respectively.

At 60 months of follow-up, lower EFS was observed in patients with large tumors (T3 or T4) (37.1% *vs* 61.6%, *p* = 0.002), with advanced nodal stage (N2 or N3) (31.2% *vs* 57.8%, *p* < 0.001), and with tumors in the BT (33.6% *vs* 50.2%, *p* = 0.01) (K–M estimates) compared with others. The significance of differences between groups remained the same in univariate Cox analysis. After multivariate Cox analysis, large tumors (T3 or T4) (HR 1.84, 95% CI 1.16–2.91, *p* = 0.009) and advanced nodal stage (N2 or N3) (HR 1.88, 95% CI 1.30–2.71, *p* = 0.001) were found to be predictors of poor EFS (Table S5 Supplement).

At the same time of follow-up, a shorter OS was observed in patients with large tumors (T3 or T4) (30.3% *vs* 64.9%, *p* = 0.001), with advanced nodal stage (N2 or N3) (29.9% *vs* 50.0%, *p* = 0.008), and with tumors in the BT (26.6% *vs* 49.1%, *p* = 0.001) (K–M estimates) compared with others. The significance of differences between groups remained the same in univariate Cox analysis. After multivariate Cox analysis, large tumors (T3 or T4) (HR 1.75, 95% CI 1.17–2.61, *p* = 0.006), advanced nodal stage (N2 or N3) (HR 1.43, 95% CI 1.04–1.97, *p* = 0.02), and tumors located on the BT (HR 1.53, 95% CI 1.11–2.10, *p* = 0.008) were found to be predictors of poor OS (Table S5 Supplement).

The SNV *ERP29* rs7114 did not influence the EFS and OS of our OPSCC patients (Table S5 Supplement).

### Survival analysis of BTSCC patients

Considering only BTSCC patients, the median follow-up was 23.0 months (range 3.4–157.1 months). At the final follow-up, 16 patients were alive without disease, 2 patients were alive with disease, and 65 patients died due to disease, and 19 patients died due to unrelated causes. The five-year EFS and OS rates were 33.6% and 26.6%, respectively.

At 60 months of follow-up, lower EFS was observed in patients with advanced nodal stage (N2 or N3) (26.2% *vs* 45.2%, *p* = 0.01) and in those with *ERP29* GG genotype (0.0% *vs* 36.2%, *p* = 0.01) (Fig. 1F) (K–M estimates) compared with others. The significance of differences between groups remained the same in univariate Cox analysis. After multivariate Cox analysis, advanced nodal stage (N2 or N3) (HR 1.77, 95% CI 1.04–2.99, *p* = 0.03) and *ERP29* GG genotype (HR 2.31, 95% CI 1.03–5.15, *p* = 0.04) were found to be a predictor of poor EFS (Table 3).

**Table 3:** Association of age, tumor characteristics and *ERP29* rs7114 genotypes with survival of 102 base of tongue squamous cell carcinoma patients in Cox analysis.

Variables	N	Univariate analysis				Multivariate analysis			
		Event-free survival		Overall survival		Event-free survival		Overall survival	
		OR (95% CI)	*p* value	OR (95% CI)	*p* value	OR (95% CI)	*p* value	OR (95% CI)	*p* value
**Age (years)**	102								
≤ 57	48	1.12 (0.69–1.82)	0.62	1.01 (0.65–1.56)	0.95	NA		NA	
> 57	54	Reference		Reference					
**Sex**	102								
Male	93	1.31 (0.52–3.28)	0.55	1.38 (0.60–3.19)	0.44	NA		NA	
Female	9	Reference		Reference					
**Histological grade**	83*								
Well or moderately	66	Reference	0.93	Reference	0.40	NA		NA	
Poorly or undifferentiated	17	1.03 (0.52–2.00)		1.29 (0.70–2.38)					
**Tumor size**	101*								
T1 or T2	14	Reference	0.10	Reference	**0.02**	Reference	0.12	Reference	**0.02**
T3 or T4	87	1.91 (0.87–4.19)		2.33 (1.12–4.84)		1.85 (0.83–4.08)		2.36 (1.13–4.92)	
**Nodal stage**	102								
N0 or N1	40	Reference	**0.01**	Reference	0.11	Reference	**0.03**	Reference	0.10
N2 or N3	62	1.85 (1.10–3.12)		1.43 (0.91–2.24)		1.77 (1.04–2.99)		1.45 (0.92–2.27)	
***ERP29 rs7114***	102								
AA	59	Reference	0.88	Reference	0.55	NA		NA	
AG or GG	43	1.03 (0.64–1.68)		1.14 (0.73–1.76)					
AA or AG	95	Reference	**0.01**	Reference	0.21	Reference	**0.04**	NA	
GG	7	2.60 (1.17–5.76)		1.64 (0.74–3.59)		2.31 (1.03–5.15)			

*n* number of patients, *OR* odds ratio, *CI* confidence interval.

*The number of patients differed from the total quoted in the survival analysis (n = 102), because it was not possible to obtain consistent information in some cases.

The significant values are indicated by bold letters.

At the same time of follow-up, a shorter OS was perceived only in patients with large tumors (T3 or T4) (20.2% *vs* 71.4%, *p* = 0.02) (K–M estimates) compared with others. The significance of differences between groups remained the same in univariate Cox analysis. After multivariate Cox analysis, large tumors (T3 or T4) (HR 2.36, 95% CI 1.13–4.92, *p* = 0.02) were found to be predictors of poor OS (Table 3). No association was found between *ERP29* rs7114 genotypes and OS.

## Discussion

We investigated whether the *ERP29* SNV rs7114 (c.*293A > G) alters the risk of OPSCC and prognosis of patients with the disease. Besides, we also investigated the role of the distinct alleles (A and G) of the referred SNV in *ERP29* expression, ERp29 protein content, miR-4421 expression and its interaction with miR-4421 in pharynx SCC cell line.

ERp29 is an ER chaperone protein and plays a role in protein maturation, secretion, and intercellular communication^5^. Although the controversial role of ERp29 in tumor development and progression^6^, ERp29 is a potential tumor suppressor in cancer^7–10^. Bambang et al. observed that ERp29 acts upregulating a group of genes with tumorous suppressive function such as E-cadherin number 1 (*CDH1*), cyclin-dependent kinase inhibitor 2B (*CDKN2B*), and spleen tyrosine kinase (*SYK*)^10^. Moreover, ERp29 downregulated a group of genes involved in cell proliferation such as epidermal growth factor receptor (*EGFR*), plasminogen activator receptor (*uPAR*), cyclin D2 (*CCND2*), and serine/threonine kinase 1 (*AKT*)^10^.

Initially, we observed that the variant *ERP29* GG genotype was more common in OPSCC patients than in controls, and that individuals with the referred genotype were under 5.67-fold increased risk of OPSCC than others. There are no studies focusing on the role of the referred SNV in the risk of OPSCC or any disease.

The *ERP29* SNV rs7114 determines the exchange of adenine (A) by guanine (G) at the 293 positions of the *ERP29* 3′-UTR, and the variant allele G creates a functional binding site for miR-4421^40,41^. miRNAs inhibit mRNA translation by directly binding to the 3′-UTR of target mRNAs, often accompanied by mRNA degradation^45^. In fact, it was already described that miRNAs are important regulatory molecules in OPSCC^46^. Overexpression of miR-4421 was associated with esophageal carcinoma development^47^. However, the role of miR-4421 in the regulation of gene expression is still unknown.

Actually, we observed that individuals carrying *ERP29* AG or GG genotypes presented lower *ERP29* expression and ERp29 protein content than individuals carrying wild-type AA genotype. Besides, individuals with AG or GG genotypes also presented higher levels of miR-4421 than individuals with the AA genotype.

Additionally, we cloned the 3′-UTR of *ERP29* into a miRNA expression vector co-transfected with miR-4421 in pharynx SCC cells (FaDu) to verify whether *ERP29* is a target gene of miR-4421, and to determine the affinity of the different alleles of the SNV rs7114 with 3′-UTR. We observed that cells co-transfected with the variant GG genotype and miR-4421 presented lower luciferase activity when compared with those co-transfected with wild-type AA genotype and miR-4421. This result indicated that miR-4421 downregulated *ERP29* expression by targeting the SNV region and, indeed, miR-4421 had more affinity with the variant G allele than with the wild-type A allele. It’s worth noting that miR-4421 is physiologically expressed in FaDu cells^48^.

All in all, our results support the fact that individuals with *ERP29* AG or GG genotypes were under increased risk of OPSCC due to *ERP29* downexpression, possibly modulated by miR-4421, and consequent loss of the tumorous suppressor function^7–10^.

We also observed that tumors located in BT and those in advanced stages were found to be predictors of poor survival, as reported in previous studies^49,50^. In fact, the OPSCC consists of a group of heterogeneous tumors with a variety of clinical characteristics^1^, thus, we performed the survival analysis only in BTSCC patients.

We observed that BTSCC patients with *ERP29* GG variant genotype had worst EFS. None of the genotypes of the studied SNV have influenced the prognosis of patients in previous studies.

Besides the increase in cell proliferation^10^, downexpression of ERp29 was associated with higher cancer cell motility and invasion^10,23^, and worst prognosis of pancreatic ductal adenocarcinoma^21^ and gastric cancer^22^ patients. In fact, ERp29 can drive MET in breast cancer cells^10^ and it may have a critical role in promoting distant metastasis during cancer progression. Furthermore, ERp29 downexpression was associated with decreased apoptosis in curcumin-treated breast cancer cells^51^ and in fibroblasts and thyrocytes from null ERp29 mice^52^.

In contrast, *ERP29* downexpression was associated with decreased RT resistance in nasopharyngeal carcinoma cells^19,20^, increased CDDP efficacy in lung cancer cell line with null p53^17^, and better prognosis of colorectal cancer patients^13^. Clearly, understanding the association of ERp29 with disease recurrence and distant metastasis is noteworthy for assessing its prognostic value in clinical applications.

Therefore, BTSCC patients carrying the *ERP29* GG variant genotype may present worst EFS due to lower ERp29 levels, leading to activation of cell proliferation, loss of cell adhesion, and MET deregulation^10^.

In summary, we identified that inheritable abnormality in *ERP29* modulates OPSCC occurrence and acts as an independent prognostic factor for EFS of BTSCC patients. We identified that *ERP29* rs7114 SNV is capable of modulating ERp29 levels, possible due to miR-4421 affinity. These findings, once validated by studies with functional protein analyses and large sample sizes, will assist in individualizing the medical care provided to patients, in which high-risk patients should receive a closer follow-up.

## Supplementary information

Supplementary Information.

## Data Availability

The authors declare that all data of the present study are available for the corresponding author upon reasonable request.
