# Supplementary material for: Role of a genetic variation in the microRNA-4421 binding site of ERP29 regarding risk of oropharynx cancer and prognosis
Source: Sci Rep. 2020 Oct 12;10:17039. doi: 10.1038/s41598-020-73675-z (PMC7550560; doi:10.1038/s41598-020-73675-z)
Supplement: Supplementary file 1 — Supplementary Information. [file 41598_2020_73675_MOESM1_ESM.docx]

**Supplementary Information**

**Role of a genetic variation in the microRNA-4421 binding site of *ERP29* regarding risk of oropharynx cancer and prognosis**

Juliana Carron^1^, Ana Paula Dalla Costa^2^, José Augusto Rinck-Junior^2^, Fernanda Viviane Mariano^3^, Benilton de Sá Carvalho^4^, Carmen Silvia Passos Lima^2^, Gustavo Jacob Lourenço^1*^

^1^Laboratory of Cancer Genetics, School of Medical Sciences, University of Campinas, Campinas, São Paulo, Brazil

^2^Department of Internal Medicine, School of Medical Sciences, University of Campinas, Campinas, São Paulo, Brazil

^3^Department of Pathology, School of Medical Sciences, University of Campinas, Campinas São Paulo, Brazil

^4^Department of Statistics, Institute of Mathematics, Statistics and Scientific Computing, University of Campinas, Campinas, São Paulo, Brazil

^*^**Corresponding author**

Gustavo Jacob Lourenço, BSc, MSc, PhD

Laboratory of Cancer Genetics, School of Medical Sciences, University of Campinas

Rua Vital Brasil, 50, Distrito de Barão Geraldo

CEP: 13083-888, Campinas, São Paulo, Brazil

Phone: +55 19 3521 9120

E-mail: guslour@unicamp.br

| **Characteristics** | **Number of patients**  **(range or %)** | **Number of controls**  **(range or %)** |
| --- | --- | --- |
| **Median age (years)** | 56 (31-85) | 44 (21-63) |
| *p-*value | **< 0.001** | |
| **Gender** |  |  |
| Male | 228 (91.2) | 228 (91.2) |
| Female | 22 (8.8) | 22 (8.8) |
| *p-*value | 1.00 | |
| **Ethnic origin** |  |  |
| White | 203 (81.2) | 203 (81.2) |
| Nonwhite | 47 (18.8) | 47 (18.8) |
| *p-*value | 1.00 | |
| **Tobacco consumption** |  |  |
| Smokers | 224 (89.6) | 33 (13.2) |
| Non-smokers | 26 (10.4) | 217 (86.8) |
| *p-*value | **< 0.001** | |
| **Alcohol consumption** |  |  |
| Drinkers | 197 (78.8) | 123 (49.2) |
| Abstainers | 53 (21.2) | 127 (50.8) |
| *p-*value | **< 0.001** | |
| **Table S1**. Demographic and smoking and alcohol habits of 250 oropharyngeal squamous cell carcinoma patients and 250 controls. | | |

| **Tumor characteristics** | **Number of patients (%)** |
| --- | --- |
| **Tumor size** |  |
| T1 | 17 (6.8) |
| T2 | 43 (17.2) |
| T3 | 70 (28.0) |
| T4 | 116 (46.4) |
| Not evaluated | 4 (1.6) |
| **Nodal stage** |  |
| N0 | 74 (29.6) |
| N1 | 38 (15.2) |
| N2 | 94 (37.6) |
| N3 | 42 (16.8) |
| Not evaluated | 2 (0.8) |
| **Distant metastasis** |  |
| M0 | 240 (96.0) |
| M1 | 3 (1.2) |
| Not evaluated | 7 (2.8) |
| **Tumor stage** |  |
| I | 9 (3.6) |
| II | 12 (4.8) |
| III | 44 (17.6) |
| IV | 182 (72.8) |
| Not evaluated | 3 (1.2) |
| **Histological grade** |  |
| Well-differentiated | 14 (5.6) |
| Moderately-differentiated | 162 (64.8) |
| Poorly-differentiated | 32 (12.8) |
| Undifferentiated | 3 (1.2) |
| Not evaluated | 39 (15.6) |
| **Tumor localization** |  |
| Base of tongue | 116 (46.4) |
| Tonsillar complex | 84 (33.6) |
| Soft palate | 45 (18.0) |
| Uvula | 3 (1.2) |
| Posterior pharyngeal wall | 2 (0.8) |
| **Table S2**. Tumor characteristics of 250 oropharynx squamous cell carcinoma patients. | |

| **Characteristics** | **Number of patients**  **(range or %)** | **Number of controls**  **(range or %)** |
| --- | --- | --- |
| **Median age (years)** | 56 (39-84) | 48 (29-58) |
| **Gender** |  |  |
| Male | 47 (95.9) | 47 (95.9) |
| Female | 2 (4.1) | 2 (4.1) |
| **Ethnic origin** |  |  |
| White | 39 (79.6) | 39 (79.6) |
| Nonwhite | 10 (20.4) | 10 (20.4) |
| **Tobacco consumption*** |  |  |
| Smokers | 47 (97.9) | 11 (22.4) |
| Non-smokers | 1 (2.1) | 38 (77.6) |
| **Alcohol consumption*** |  |  |
| Drinkers | 46 (97.9) | 29 (59.2) |
| Abstainers | 1 (2.1) | 20 (40.8) |
| **Tumor stage*** |  |  |
| I | 1 (2.1) | NA |
| II | 5 (10.4) | NA |
| III | 7 (14.6) | NA |
| IV | 35 (72.9) | NA |
| **Histological grade*** |  |  |
| Well-differentiated | 3 (6.8) | NA |
| Moderately-differentiated | 34 (77.3) | NA |
| Poorly-differentiated | 6 (13.6) | NA |
| Undifferentiated | 1 (2.3) | NA |
| **Table S3**. Clinical and pathological characteristics of the 49 base of tongue squamous cell carcinoma patients and 49 controls included in the stage 1 analysis. NA: not applicable. *The number of patients differed from the total quoted in the analysis (n= 49), because it was not possible to obtain consistent information in some cases. | | |

| **SNV** | **Gene** | **Chromosome** | **Gene function*** | **Type of cancer*** | **Sample size**** | **MicroRNA binding***** |
| --- | --- | --- | --- | --- | --- | --- |
| rs7930 | *TOMM20* | 1q42 | Apoptosis | Gastric^1^, colorectal^2^ | 833 | Yes |
| rs7917618 | *CCDC6* | 10q21 | Cell cycle | Lung^3^, bladder^4^ | 1503 | Yes |
| rs16925538 | *MPPED2* | 11p13 | Cell proliferation | Neuroblastoma^5^, oral^6^, breast^7^ | 2132 | Yes |
| rs4758909 | *CHFR* | 12q24.33 | Cell cycle | Gastric^8^, lung^9^ | 908 | Yes |
| rs4900833 | *MBIP* | 14q13.3 | Apoptosis | Lung^10^, breast^11^ | 565 | Yes |
| rs4243226 | *PLCG2* | 16q24.1 | Cell signing | Head and neck^12^, leukemia^13^ | 419 | No |
| rs2240774 | *JMJD6* | 17q25 | Cell cycle | Colon^14^, head and neck^15^, breast^16^ | 107 | Yes |
| rs11412 | *FLNB* | 3p14.3 | Cell adhesion | Melanoma^17^, breast^18^ | 1430 | Yes |
| rs1131312 | *FLNB* | 3p14.3 | Cell adhesion | Melanoma^17^, breast^18^ | 1766 | Yes |
| rs2370512 | *SLCO2A1* | 3q21 | Prostaglandin | Head and neck^19^, colon^20^ | 180 | Yes |
| rs7674870 | *SLC7A11* | 4q28.3 | Cell proliferation | Head and neck^21^, bladder^22^ | 101 | Yes |
| rs17796864 | *SH3RF2* | 5q32 | Apoptosis | Colon^23^ | 960 | No |
| rs6914716 | *MYO6* | 6q13 | Cell proliferation | Liver^24^, oral^25^ | 123 | Yes |
| rs1128957 | *TUSC1* | 9p21.2 | Cell proliferation | Lung^26^, gastric^27^, glioblastoma^28^ | 65 | Yes |
| rs7114 | *ERP29* | 12q24 | Unfolded protein response | Breast^29,30^, colorectal^31^ | 234 | Yes |
| rs1736078 | *CTSB* | 8p22 | Intracellular proteolysis | Breast^32^, glioblastoma^33^ | 1195 | Yes |
| **Table S4**. Single nucleotide variations located at 3’-untranslated region related to genes of carcinogenesis pathways and microRNA *in silico* analysis. (SNV) single nucleotide variation, (rs) SNV reference number. *According to *Database for Annotation, Visualization and Integrated Discovery* (DAVID) program^34,35^ and *National Center for Biotechnology Information* (NCBI) database^36^. **Sample size calculated based on the genotypic frequencies observed in healthy individuals from different ethnic populations^37^. ****In silico* analysis according to MicroSNiPer^38^ and MirSNPscore^39^ algorithms. | | | | | | |

| **Variables** | **n** | **Univariate analysis** | | | |  | **Multivariate analysis** | | | | | |
| --- | --- | --- | --- | --- | --- | --- | --- | --- | --- | --- | --- | --- |
|  |  | **Event-free survival** | | **Overall survival** | |  | **Event-free survival** | | | **Overall survival** | | |
|  |  | **OR**  **(95% CI)** | ***p* value** | **OR**  **(95% CI)** | ***p* value** | **a** | **OR**  **(95% CI)** | | ***p***  **value** | **OR**  **(95% CI)** | | ***p***  **value** |
| **Age (years)** | 226 |  |  |  |  |  |  | |  |  | |  |
| ≤ 57 | 118 | 1.24 (0.87-1.75) | 0.21 | 1.08 (0.79-1.48) | 0.60 |  | NA | | | NA | | |
| > 57 | 108 | Reference |  | Reference |  |  |  |  |  |  |  |  |
| **Gender** | 226 |  |  |  |  |  |  |  | |  |  | |
| Male | 205 | 1.48 (0.75-2.92) | 0.25 | 1.46 (0.81-2.63) | 0.20 |  | NA | | | NA | | |
| Female | 21 | Reference |  | Reference |  |  |  |  |  |  |  |  |
| **Histological grade** | 190* |  |  |  |  |  |  |  | |  |  | |
| Well or moderately | 157 | Reference | 0.81 | Reference | 0.98 |  | NA | | | NA | | |
| Poorly or undifferentiated | 33 | 1.06 (0.64-1.74) |  | 1.00 (0.64-1.56) |  |  |  |  |  |  |  |  |
| **Tumor size** | 225* |  |  |  |  |  |  | |  |  | |  |
| T1 or T2 | 55 | Reference | **0.002** | Reference | **0.001** |  | Reference | | **0.009** | Reference | | **0.006** |
| T3 or T4 | 170 | 2.03 (1.29-3.20) |  | 1.97 (1.32-2.92) |  |  | 1.84 (1.16-2.91) | |  | 1.75 (1.17-2.61) | |  |
| **Nodal stage** | 226 |  |  |  |  |  |  | |  |  | |  |
| N0 or N1 | 99 | Reference | **< 0.001** | Reference | **0.009** |  | Reference | | **0.001** | Reference | | **0.02** |
| N2 or N3 | 127 | 1.97 (1.36-2.83) |  | 1.53 (1.11-2.10) |  |  | 1.88 (1.30-2.71) | |  | 1.43 (1.04-1.97) | |  |
| **Tumor localization** | 226 |  |  |  |  |  |  | |  |  | |  |
| Base of tongue | 102 | 1.53 (1.08-2.17) | **0.01** | 1.73 (1.26-2.36) | **0.001** |  | 1.34 (0.94-1.91) | | 0.10 | 1.53 (1.11-2.10) | | **0.008** |
| Others | 124 | Reference |  | Reference |  |  | Reference | |  | Reference | |  |
| ***ERP29* rs7114** | 226 |  |  |  |  |  |  | |  |  | |  |
| AA | 134 | Reference | 0.68 | Reference | 0.46 |  | NA | | | NA | | |
| AG or GG | 92 | 1.07 (0.76-1.52) |  | 1.12 (0.82-1.54) |  |  |  |  |  |  |  |  |
| AA or AG | 211 | Reference | 0.22 | Reference | 0.65 |  | NA | | | NA | | |
| GG | 15 | 1.46 (0.79-2.72) |  | 1.14 (0.63-2.06) |  |  |  |  |  |  |  |  |
| **Table S5**. Association of age, tumor characteristics and *ERP29* rs7114 genotypes with survival of 226 oropharynx squamous cell carcinoma patients in Cox analysis. n: number of patients, OR: odds ratio, CI: confidence interval. *The number of patients differed from the total quoted in the survival analysis (n= 101), because it was not possible to obtain consistent information in some cases. The significant values are indicated by bold letters. | | | | | | | | | | | | |

**References**

1. Zhao, Z. *et al*. Stromal-epithelial metabolic coupling in gastric cancer: stromal MCT4 and mitochondrial TOMM20 as poor prognostic factors. *Eur. J. Surg. Oncol.* **40**(10), 1361-1368 (2014).

2. Park, S. H. *et al*. TOMM20 as a potential therapeutic target of colorectal cancer. *BMB Rep*. **52**(12), 712-717 (2019).

3. Matsubara, D. *et al*. Identification of CCDC6-RET fusion in the human lung adenocarcinoma cell line, LC-2/ad. *J. Thorac. Oncol.* **7**(12), 1872-1876 (2012).

4. Morra, F. *et al*. CCDC6 and USP7 expression levels suggest novel treatment options in high-grade urothelial bladder cancer. *J. Exp. Clin. Cancer Res*. **38**(1), 90 (2019).

5. Liguori, L. *et al*. The metallophosphodiesterase Mpped2 impairs tumorigenesis in neuroblastoma. *Cell Cycle* **11**(3), 569-581 (2012).

6. Shen, L. *et al*. miR-448 downregulates MPPED2 to promote cancer proliferation and inhibit apoptosis in oral squamous cell carcinoma. *Exp. Ther. Med*. **12**(4), 2747-2752 (2016).

7. Pellecchia, S. *et al*. The Metallophosphoesterase-Domain-Containing Protein 2 (MPPED2) Gene Acts as Tumor Suppressor in Breast Cancer. *Cancers (Basel)* **11**(6), 797 (2019).

8. Wang, M., Shen, L. & Deng, D. Association between CHFR methylation and chemosensitivity of paclitaxel in advanced gastric cancer. *Med. Oncol*. **31**(4), 907 (2014).

9. Wang, C. *et al*. Clinicopathological significance of CHFR methylation in non-small cell lung cancer: a systematic review and meta-analysis. *Oncotarget* **8**(65), 109732-109739 (2017).

10. Harris, T. *et al*. Both gene amplification and allelic loss occur at 14q13.3 in lung cancer. *Clin. Cancer Res*. **17**(4), 690-699 (2011).

11. Lee, Y. H., Kim, J. H. & Song, G. G. Genome-wide pathway analysis of breast cancer. *Tumour Biol*. **35**(8), 7699-7705 (2014).

12. Gyanchandani, R. *et al*. A proangiogenic signature is revealed in FGF-mediated bevacizumab-resistant head and neck squamous cell carcinoma. *Mol. Cancer Res.* **11**(12), 1585-1596 (2013).

13. Jones, D. *et al*. PLCG2 C2 domain mutations co-occur with BTK and PLCG2 resistance mutations in chronic lymphocytic leukemia undergoing ibrutinib treatment. *Leukemia* **31**(7), 1645-1647 (2017).

14. Wang, F. *et al*. JMJD6 promotes colon carcinogenesis through negative regulation of p53 by hydroxylation. *PLoS Biol*. **12**(3), e1001819 (2014).

15. Guo, B. *et al*. Jumonji domain-containing protein 6 functions as a marker of head and neck squamous cell carcinoma at advanced stage with no effect on prognosis. *Oncol. Lett*. **18**(6), 5843-5852 (2019).

16. Nandy, D., Rajam, S. M. & Dutta, D. A three layered histone epigenetics in breast cancer metastasis. *Cell Biosci*. **10**, 52 (2020).

17. Bandaru, S. *et al*. Targeting filamin B induces tumor growth and metastasis via enhanced activity of matrix metalloproteinase-9 and secretion of VEGF-A. *Oncogenesis* **3**, e119 (2014).

18. Li, J. *et al*. An alternative splicing switch in FLNB promotes the mesenchymal cell state in human breast cancer. *Elife* **7**, e37184 (2018).

19. Zolk, O. *et al*. Transporter gene expression in human head and neck squamous cell carcinoma and associated epigenetic regulatory mechanisms. *Am. J. Pathol*. **182**(1), 234-243 (2013).

20. Guda, K. *et al*. Inactivating mutation in the prostaglandin transporter gene, SLCO2A1, associated with familial digital clubbing, colon neoplasia, and NSAID resistance. *Cancer Prev. Res. (Phila)* **7**(8), 805-812 (2014).

21. Yoshikawa, M. *et al*. xCT inhibition depletes CD44v-expressing tumor cells that are resistant to EGFR-targeted therapy in head and neck squamous cell carcinoma. *Cancer Res*. **73**(6), 1855-1866 (2013).

22. Drayton, R. M. *et al*. Reduced expression of miRNA-27a modulates cisplatin resistance in bladder cancer by targeting the cystine/glutamate exchanger SLC7A11. *Clin. Cancer Res*. **20**(7), 1990-2000 (2014).

23. Kim, J. C. *et al*. Novel single-nucleotide polymorphism markers predictive of pathologic response to preoperative chemoradiation therapy in rectal cancer patients. *Int. J. Radiat. Oncol. Biol. Phys*. **86**(2), 350-357 (2013).

24. Ma, X. *et al*. Knockdown of Myosin VI Inhibits Proliferation of Hepatocellular Carcinoma Cells In Vitro. *Chem. Biol. Drug Des*. **86**(4), 723-730 (2015).

25. Zhang, X. *et al*. Knockdown of Myosin 6 inhibits proliferation of oral squamous cell carcinoma cells. *J. Oral Pathol. Med*. **45**(10), 740-745 (2016).

26. Shan, Z. *et al*. TUSC1, a putative tumor suppressor gene, reduces tumor cell growth in vitro and tumor growth in vivo. *PLoS One* **8**(6), e66114 (2013).

27. Kanda, M. *et al*. Clinical significance of expression and epigenetic profiling of TUSC1 in gastric cancer. *J. Surg. Oncol*. **110**(2), 136-144 (2014).

28. Zhang, R. *et al*. Tumor Suppressor Candidate 1 Suppresses Cell Growth and Predicts Better Survival in Glioblastoma. *Cell Mol. Neurobiol*. **37**(1), 37-42 (2017).

29.Bambang, I. F. *et al*. Endoplasmic reticulum protein 29 regulates epithelial cell integrity during the mesenchymal-epithelial transition in breast cancer cells. *Oncogene* **32**(10), 1240-1251 (2013).

30. Chen, S., Zhang, Y. & Zhang, D. Endoplasmic reticulum protein 29 (ERp29) confers radioresistance through the DNA repair gene, O(6)-methylguanine DNA-methyltransferase, in breast cancer cells. *Sci. Rep*. **5**, 14723 (2015).

31. Deng, Y. J. *et al*. CLIC4, ERp29, and Smac/DIABLO derived from metastatic cancer stem-like cells stratify prognostic risks of colorectal cancer. *Clin. Cancer Res*. **20**(14), 3809-3817 (2014).

32. Zubor, P. *et al*. Gene expression abnormalities in histologically normal breast epithelium from patients with luminal type of breast cancer. *Mol. Biol. Rep*. **42**(5), 977-988 (2015).

33. Ho, K. H. *et al*. miR-140 targeting CTSB signaling suppresses the mesenchymal transition and enhances temozolomide cytotoxicity in glioblastoma multiforme. *Pharmacol. Res*. **147**, 104390 (2019).

34. Huang, D. W., Sherman, B. T. & Lempicki, R. A. Bioinformatics enrichment tools: paths toward the comprehensive functional analysis of large gene lists. *Nucleic Acids Res*. **37**(1), 1-13 (2009).

35. Huang, D. W., Sherman, B. T. & Lempicki, R. A. Systematic and integrative analysis of large gene lists using DAVID bioinformatics resources. *Nat. Protoc*. **4**(1), 44-57 (2009).

36. Geer, L. Y. *et al*. The NCBI BioSystems database. *Nucleic Acids Res*. **38**, D492-6 (2010).

37. International Hapmap 3 Consortium *et al*. Integrating common and rare genetic variation in diverse human populations. *Nature* **467**(7311), 52-58 (2010).

38. Barenboim, M., Zoltick, B. J., Guo, Y. & Weinberger, D. R. MicroSNiPer: a web tool for prediction of SNP effects on putative microRNA targets. *Hum. Mutat.* **31**(11), 1223-1232 (2010).

39. Thomas, L. F., Saito, T. & Sætrom, P. Inferring causative variants in microRNA target sites. *Nucleic Acids Res.* **39**(16), e109 (2011).
